# Supplementary material for: Fungal Als proteins hijack host death effector domains to promote inflammasome signaling
Source: Nat Commun. 2025 Feb 12;16:1562. doi: 10.1038/s41467-025-56657-5 (PMC11821908; doi:10.1038/s41467-025-56657-5)
Supplement: Supplementary file 1 — Supplementary Information [file 41467_2025_56657_MOESM1_ESM.pdf]

Fungal Als proteins hijack host death effector domains to promote inflammasome signaling

Tingting Zhou[1], Norma V Solis[2], Michaela Marshall[3], Qing Yao[4†], Eric Pearlman[3], Scott G Filler[2,5], Haoping Liu[1]*

[1]Department of Biological Chemistry, University of California; Irvine, CA, 92697, USA.

[2]Division of Infectious Diseases, Lundquist Institute for Biomedical Innovation at Harbor-UCLA Medical Center; Torrance, CA, 90502, USA.

[3]Department of Physiology and Biophysics, University of California; Irvine, CA, 92697, USA.

[4]Division of Biology and Biological Engineering, California Institute of Technology; Pasadena, CA, 91125, USA.

[5]David Geffen School of Medicine at UCLA; Los Angeles, CA, 90024, USA.

[†] Present address: Gilead Sciences Inc; Foster City, CA, 94404, USA.

[*]Corresponding author. Email: h4liu@uci.edu

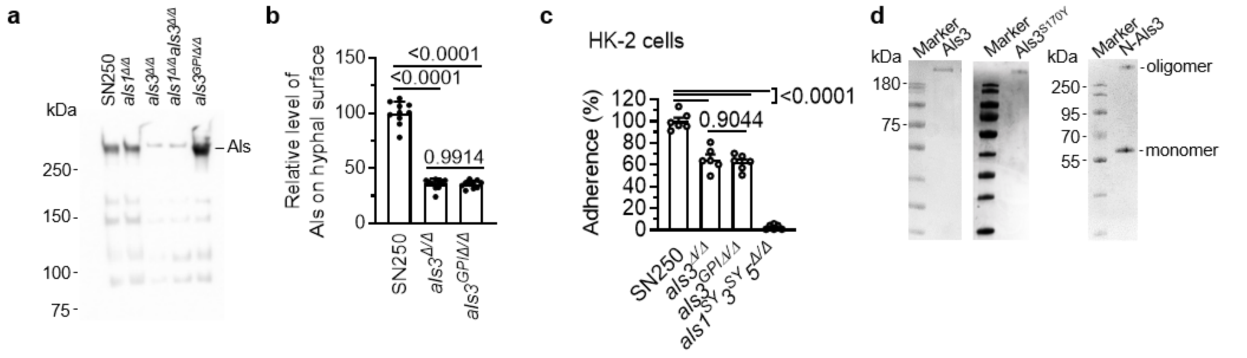

Supplementary Fig. 1. The released Als3 induces inflammatory responses. a, The released Als protein in the supernatant of the indicated *C. albicans* hyphal cultures was determined by Western blotting. Results are representative of three individual experiments. **b**, Relative level of the fluorescent intensity of immunostaining images for Als proteins. The average values of 10 different areas for strain from two independent experiments were analyzed with Leica LAS AF Lite software. *P* values were calculated using one-way ANOVA with Tukey post-hoc analysis. **c**, Different *als* mutants were tested for their ability to adhere to human kidney-2 (HK-2) cells. Data are mean ± s.e.m. from two experiments. *P* values were calculated using one-way ANOVA with Tukey post-hoc analysis (n=6). **d**, 5 μg purified Als3, Als3^{S170Y}, or N-Als3 on SDS-PAGE gels.

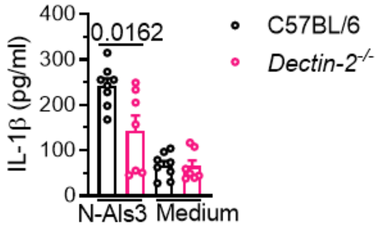

Supplementary Fig. 2. Als3 induces inflammatory responses partially through Dectin-2. ELISA analysis of secreted IL-1β from BMDCs after stimulation with purified N-Als3 (5.3 µg ml^{-1}) for 24 h. Data are mean ± s.e.m. from three independent experiments. *P* values were calculated using an unpaired two-tailed *t*-test (n=8 for C57BL/6, n=7 for *Dectin-2*$^{-/-}$).

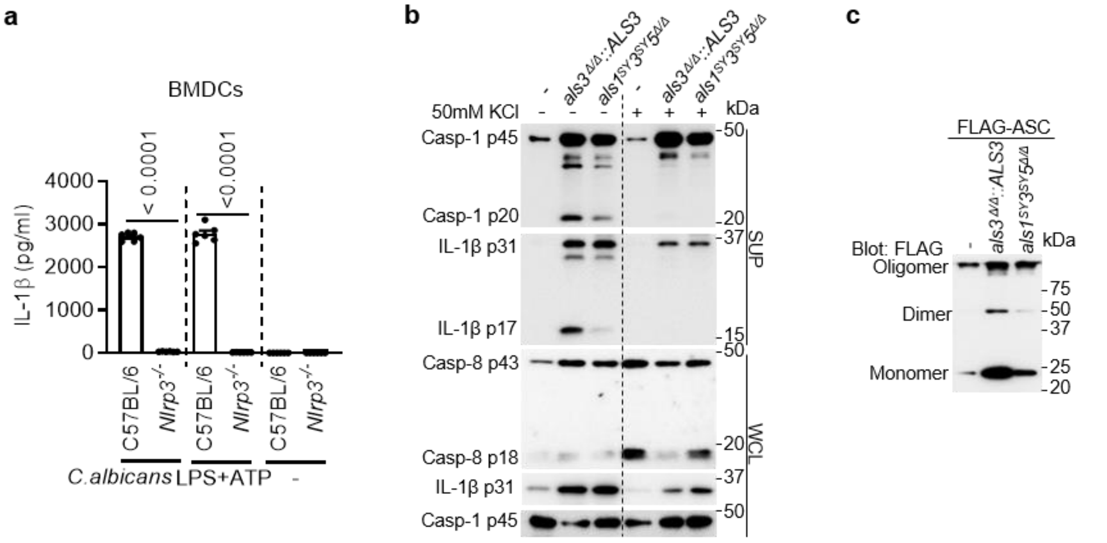

Supplementary Fig. 3. Internalized Als proteins mediate inflammasome activation. a, BMDCs from wild-type or *Nlrp3*$^{-/-}$ mice were stimulated with *C. albicans* yeasts for 24 h or ATP (5 mM) for 2 h after LPS prestimulation (0.5 ng ml^{-1} for 3 h). *P* values were calculated with an unpaired two-tailed t-test (n=6 from two experiments). The experiment was repeated at least three times with similar trends. Data are mean ± s.e.m.. **b**, Processing of caspase-1 (p10), caspase-8 (p18), and IL-1β (p17) was determined by Western blotting after infection with the indicated *C. albicans* strains in the presence or absence of 50 mM KCl. **c**, Western blot analysis of WT 293T cells with the overexpression of FLAG-tagged mouse ASC (FLAG–ASC) following infection with the *als3*$^{\Delta/\Delta}$:*ALS3* strain or the *als1*^{SY}*3*^{SY}*5*$^{\Delta/\Delta}$ mutant for 3 h. Samples were cross-linked with DSS. Results in (**b**, **c**) are representative of three biologically independent experiments.

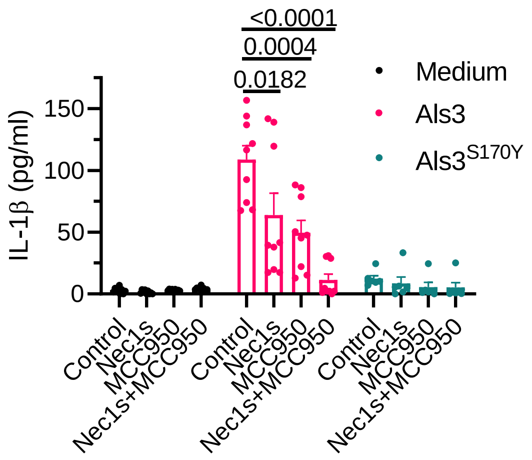

Supplementary Fig. 4. Nec1s and MCC950 inhibited Als3-induced IL-1β in BMDCs. ELISA analysis of secreted IL-1β from BMDCs after stimulation with purified Als3 (5 µg ml^{-1}) or $Als3^{S170Y}$ (5 µg ml^{-1}) for 24 h in the presence of Nec-1s (10 µM) and/or MCC950 (10 µM). Data are mean ± s.e.m.. from three independent experiments for medium (n=9) and Als3 (n=9) and from two independent experiments for $Als3^{S170Y}$ (n=6). *P* values were calculated using one-way ANOVA with Tukey post-hoc analysis.

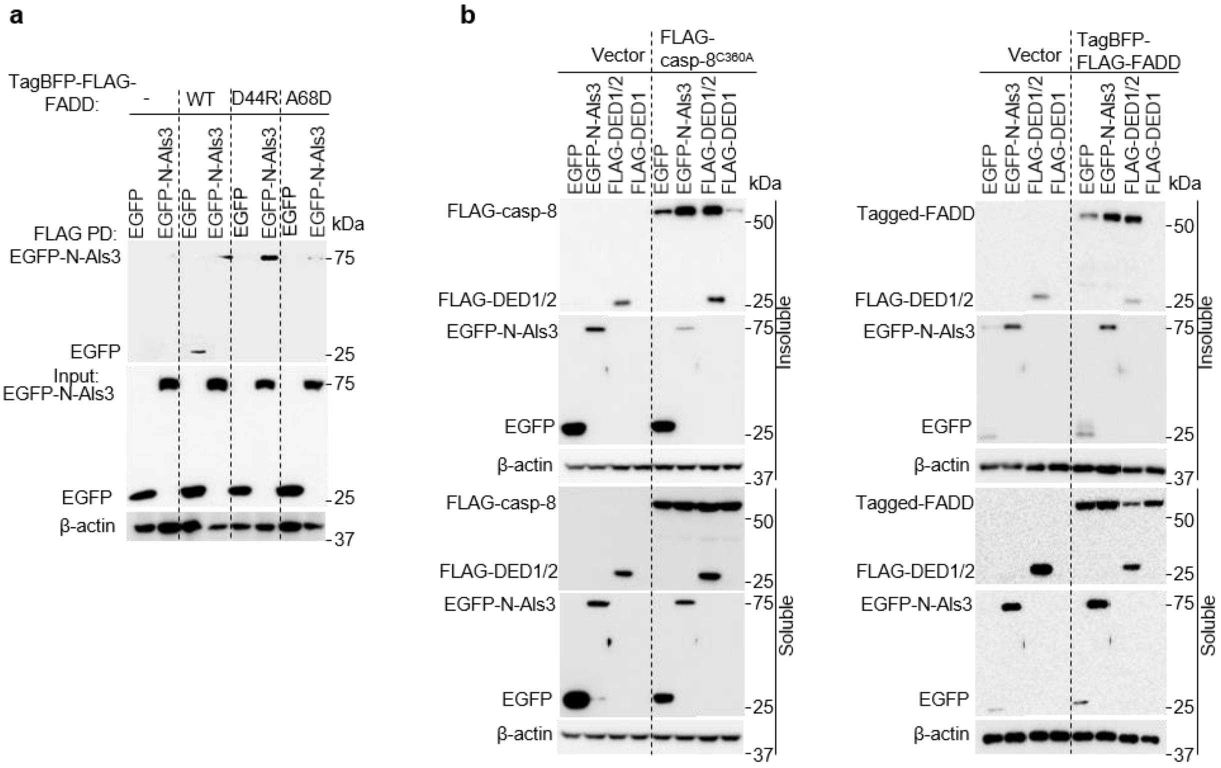

Supplementary Fig. 5. Als3 promotes the oligomerization of caspase-8 and FADD, resembling caspase-8 DED1/2. a, Immunoprecipitation of *Casp8*$^{-/-}$ 293T lysates overexpressing empty vector (−), tagBFP-FLAG-tagged human FADD, FADD^{D44R}, or FADD^{A68D} with EGFP-N-Als3. Soluble fractions were used for pull-down with anti-FLAG magnetic beads. **b**, Western blot analysis of *Casp8*$^{-/-}$ 293T cells transfected with EGFP-N-Als3, human caspase-8 DED1/2, or DED1 together with FLAG-caspase-8^{C360A} or tagBFP-FLAG-FADD. All results are representative of three individual experiments.

Strain	Parental strain	guide RNA	Donor DNA	Restriction enzyme for point mutation identification
als3$^{GPI\Delta/\Delta}$	SN250 (arg+)	CTTACTACATTTGACG GATC	TTCATCTTTAGTTAGTTTACATATGCTTACTAC ATTTGACGGATCCTAAGGATGATACCATCATA GTCGCCTTTTTAGATTTTTGTTATT	
als3^{V309N}	SN250 (arg+)	ATCTAACGGTATTGTT ATTG	GGATACAGAAATAGTGATGCTGGATCTAACGG TATTAATATTGTCGCTACTACCAGAACAGTTAC AGACAGTACTACCGCTGTGACCACC	VspI
als3$^{C\text{-}Als3\Delta/\Delta}$::7x His	SN250 (arg+)	(same as the gRNA used to creat *als3*$^{GPI\Delta/\Delta}$)	TCCAACTGACTCAATAGACACTGTCATTGTACA AGTTCCA CACCACCATCACCATCATCAC TAAGGATGATACCATCATAGTCGCCTTTTTAGA TTTTTGTTATTT	
als3$^{S170YC\text{-}Als3\Delta/\Delta}$::7x His	SN250 (arg+)	(same as the gRNA used to creat *als3*$^{GPI\Delta/\Delta}$)	(same as the donor DNA used to creat #3 strain)	

Supplementary Table 1. ***C. albicans*** **strains created used in this research.** Each gene editing was done for two copies.

Antibodies	dilutions	company names	catalog numbers	clone numbers (monoclonal)	Application
anti-IL-1β	1:2000	R&D Systems	AF-401-NA	N/A	Western Blotting(WB)
anti-caspase-1	1:1000	AdipoGen	AG-20B-0042--C100	Casper-1	WB
anti-GSDMD	1:1000	Abcam	ab209845	EPR19828	WB
anti-β-actin-HRP	1:5000	CST	5125S	13E5	WB
anti-FLAG-HRP	1:3000	CST	86861S	D6W5B	WB
anti-GFP-HRP	1:3000	CST	2037S	D5.1	WB
anti-caspase-8 mouse-specific	1:1000	Enzo	ALX-804-447-C100	1G12	WB
	1:100				Immunofluorescence (IF)
anti-cleaved-caspase-8 mouse-specific	1:500	CST	9429S	Asp387	WB
anti-caspase-8 human-specific	1:1000	CST	9746S	1C12	WB
anti-FADD	1:500	SCBT	sc-271748	G-4	WB
anti-RIPK1	1:1000	CST	3493S	D94C12	WB
anti-ASC	1:1000	CST	67824S	D2W8U	WB
anti-rabbit-HRP	1:7500	Jackson Immuno Research Laboratories	111-035-003	N/A	WB
anti-mouse-HRP	1:7500	Jackson Immuno Research Laboratories	115-035-003	N/A	WB
anti-goat-HRP	1:7500	Jackson Immuno Research Laboratories	705-035-003	N/A	WB
anti-Als	1: 250	Dr. Scott. Filler's Lab (originally from PickCell Laboratories, http://www.pickcell-b2b.com)		N/A	WB
	1: 50				IF
anti-ASC	1:100	Millipore	04-147		IF
anti-EEA1	1:200	CST	3288T	C45B10	IF
FITC-conjugated anti-mouse	1:100	Jackson ImmunoResearch	115-095-003	N/A	IF
AlexaFluor568-conjugated anti-rat	1:100	Invitrogen	**A-11077**	N/A	IF

AlexaFluor647-conjugated anti-rabbit	1:100	Invitrogen	A-31573	N/A	IF
AlexaFluor488-conjugated anti-rabbit	1:100	Invitrogen	A-11008	N/A	IF

Supplementary Table 2. Antibodies used in this research.
