# Supplementary material for: Fungal Als proteins hijack host death effector domains to promote inflammasome signaling
Source: Nat Commun. 2025 Feb 12;16:1562. doi: 10.1038/s41467-025-56657-5 (PMC11821908; doi:10.1038/s41467-025-56657-5)
Supplement: Supplementary file 2 — Reporting Summary [file 41467_2025_56657_MOESM2_ESM.pdf]

nature portfolio

Corresponding author(s): Haoping Liu

Last updated by author(s): Jan 4, 2025

# Reporting Summary

Nature Portfolio wishes to improve the reproducibility of the work that we publish. This form provides structure for consistency and transparency in reporting. For further information on Nature Portfolio policies, see our Editorial Policies and the Editorial Policy Checklist.

## Statistics

For all statistical analyses, confirm that the following items are present in the figure legend, table legend, main text, or Methods section.

| n/a | Confirmed | |
|---|---|---|
| ☐ | ☒ | The exact sample size ($n$) for each experimental group/condition, given as a discrete number and unit of measurement |
| ☐ | ☒ | A statement on whether measurements were taken from distinct samples or whether the same sample was measured repeatedly |
| ☐ | ☒ | The statistical test(s) used AND whether they are one- or two-sided<br>*Only common tests should be described solely by name; describe more complex techniques in the Methods section.* |
| ☐ | ☒ | A description of all covariates tested |
| ☐ | ☒ | A description of any assumptions or corrections, such as tests of normality and adjustment for multiple comparisons |
| ☐ | ☒ | A full description of the statistical parameters including central tendency (e.g. means) or other basic estimates (e.g. regression coefficient) AND variation (e.g. standard deviation) or associated estimates of uncertainty (e.g. confidence intervals) |
| ☐ | ☒ | For null hypothesis testing, the test statistic (e.g. $F$, $t$, $r$) with confidence intervals, effect sizes, degrees of freedom and $P$ value noted<br>*Give P values as exact values whenever suitable.* |
| ☒ | ☐ | For Bayesian analysis, information on the choice of priors and Markov chain Monte Carlo settings |
| ☒ | ☐ | For hierarchical and complex designs, identification of the appropriate level for tests and full reporting of outcomes |
| ☒ | ☐ | Estimates of effect sizes (e.g. Cohen's $d$, Pearson's $r$), indicating how they were calculated |

*Our web collection on statistics for biologists contains articles on many of the points above.*

## Software and code

Policy information about availability of computer code

| | |
|---|---|
| Data collection | Imaging was done by Leica SP8. |
| Data analysis | Imaging was analyzed with Image J (1.51j8) and Leica LAS AF software. GraphPad Prism software 9.5.1 was used for statistic analysis. |

For manuscripts utilizing custom algorithms or software that are central to the research but not yet described in published literature, software must be made available to editors and reviewers. We strongly encourage code deposition in a community repository (e.g. GitHub). See the Nature Portfolio guidelines for submitting code & software for further information.

## Data

Policy information about availability of data

All manuscripts must include a data availability statement. This statement should provide the following information, where applicable:

- Accession codes, unique identifiers, or web links for publicly available datasets
- A description of any restrictions on data availability
- For clinical datasets or third party data, please ensure that the statement adheres to our policy

The data supporting the findings from this study are available within the article file and its supplementary information. Any other raw data or noncommercial material used in this study are available from the corresponding author upon reasonable request.

## Research involving human participants, their data, or biological material

Policy information about studies with human participants or human data. See also policy information about sex, gender (identity/presentation), and sexual orientation and race, ethnicity and racism.

| | |
|---|---|
| Reporting on sex and gender | No human participants or human data are used in this study. |
| Reporting on race, ethnicity, or other socially relevant groupings | No human participants or human data are used in this study. |
| Population characteristics | No human participants or human data are used in this study. |
| Recruitment | No human participants or human data are used in this study. |
| Ethics oversight | No human participants or human data are used in this study. |

Note that full information on the approval of the study protocol must also be provided in the manuscript.

# Field-specific reporting

Please select the one below that is the best fit for your research. If you are not sure, read the appropriate sections before making your selection.

[x] Life sciences [ ] Behavioural & social sciences [ ] Ecological, evolutionary & environmental sciences

For a reference copy of the document with all sections, see nature.com/documents/nr-reporting-summary-flat.pdf

# Life sciences study design

All studies must disclose on these points even when the disclosure is negative.

| | |
|---|---|
| Sample size | The exact n for each experiment was described in corresponding figure legends. Sample sizes were determined based on expected effect size and variability within the sample, previous experience and standards in the field. Low variability between the same type of samples, indicated as SME, confirming that n=3-6 samples is sufficient to observe statistically significant differences between relevant groups. |
| Data exclusions | No data was excluded from analysis. |
| Replication | At least two independent experiments were performed for each experiments, each panel presented the representative data or combined data. All reported data were reproduced reliably. |
| Randomization | For animal models, all mice used in this study were divided into different treatment groups randomly. For cell culture, cells were divided into each plates and assigned into different treatments groups randomly. |
| Blinding | All data acquisition and analysis in this study were performed in a blinded way. |

# Reporting for specific materials, systems and methods

We require information from authors about some types of materials, experimental systems and methods used in many studies. Here, indicate whether each material, system or method listed is relevant to your study. If you are not sure if a list item applies to your research, read the appropriate section before selecting a response.

### Materials & experimental systems

| n/a | Involved in the study | |
|---|---|---|
| [ ] | [x] | Antibodies |
| [ ] | [x] | Eukaryotic cell lines |
| [x] | [ ] | Palaeontology and archaeology |
| [ ] | [x] | Animals and other organisms |
| [x] | [ ] | Clinical data |
| [x] | [ ] | Dual use research of concern |
| [x] | [ ] | Plants |

### Methods

| n/a | Involved in the study | |
|---|---|---|
| [x] | [ ] | ChIP-seq |
| [x] | [ ] | Flow cytometry |
| [x] | [ ] | MRI-based neuroimaging |

## Antibodies

| | |
|---|---|
| Antibodies used | All antibodies were listed in the methods and supplementary table 2. |
| Validation | All antibodies and reagents were purchased from well-established and reputable companies, all of which have ISO certification. |

All antibodies were validated before use. This involves appropriate negative and positive control.
Detailed validation from manufacturer's data sheets:
anti-IL-1β (# AF-401-NA; Recommended concentration 0.25 μg/mL, Validate for WB) https://www.rndsystems.com/products/mouse-il-1beta-il-1f2-antibody_af-401-na#product-details

anti-caspase-1(# AG-20B-0042--C100; Recommended concentration 1μg/ml, Validate for WB)
https://adipogen.com/ag-20b-0042-anti-caspase-1-p20-mouse-mab-casper-1.html

anti-GSDMD (# ab209845; Recommended concentration 1/1000, Validate for WB)
https://www.abcam.com/products/primary-antibodies/gsdmd-antibody-epr19828-ab209845.html

anti-β-actin-HRP (#5125S; Recommended concentration 1:1000, Validate for WB)
https://www.cellsignal.com/products/antibody-conjugates/b-actin-13e5-rabbit-mab-hrp-conjugate/5125

anti-FLAG-HRP (#86861S; Recommended concentration 1/1000, Validate for WB)
https://www.cellsignal.com/products/antibody-conjugates/dykddddk-tag-d6w5b-rabbit-mab-binds-to-same-epitope-as-sigma-aldrich-anti-flag-m2-antibody-hrp-conjugate/86861?srsltid=AfmBOorQVwtyv-wDrcMXr1Uts1sZ1yTkm5KHTYpig5kSaouBB2lIWd9v

anti-GFP-HRP (#2037S; Recommended concentration 1/1000, Validate for WB)
https://www.cellsignal.com/products/antibody-conjugates/gfp-d5-1-rabbit-mab-hrp-conjugate/2037

anti-caspase-8 mouse-specific (#ALX-804-447-C100; Validate for WB)
https://www.enzo.com/product/caspase-8-mouse-monoclonal-antibody-1g12/#pictures

anti-cleaved-caspase-8 mouse-specific (#9429S; Recommended concentration 1/1000, Validate for WB)
https://www.cellsignal.com/products/primary-antibodies/cleaved-caspase-8-asp387-antibody/9429?srsltid=AfmBOorgmD00XQDs-6oWLBIAq_tzbusPmamgszGDGIJHXZ_yWyWOuKBK

anti-caspase-8 human-specific (#9746S; Recommended concentration 1/1000, Validate for WB)
https://www.cellsignal.com/products/primary-antibodies/caspase-8-1c12-mouse-mab/9746?srsltid=AfmBOoqx4Y-ledQfF6L1_MnB-ufRl7F7S1xBkjA_ubOW4Tllllbeeuzd

anti-FADD (#sc-271748; Validate for WB)
https://www.scbt.com/p/fadd-antibody-g-4?srsltid=AfmBOoqOzpXRW4_VF72lDyra43-LjwMvmoy9EtbMdM_CwSTheIuUCvqH

anti-RIPK1(#3493S; Recommended concentration 1/1000, Validate for WB)
https://www.cellsignal.com/products/primary-antibodies/rip-d94c12-xp-rabbit-mab/3493?srsltid=AfmBOorMfvxpGJaGTFLp0u3zU3VZ0-0Mc1SzhPR9H9BIpYalguWqpiTP

anti-ASC (#67824S; Recommended concentration 1/1000, Validate for WB)
https://www.cellsignal.com/products/primary-antibodies/asc-tms1-d2w8u-rabbit-mab/67824

anti-caspase-8 (#ALX-804-447-C100; Validate for Immunohistochemistry and WB)
https://www.enzo.com/product/caspase-8-mouse-monoclonal-antibody-1g12/

anti-Als (Dr. Scott. Filler's Lab, originally from PickCell Laboratories, http://www. pickcell-b2b.com; validated for IF)
IF application was published in PMID: 17311474. WB application was confirmed in this paper by comparing WT and als mutants (Supplementary Fig. 1a).

anti-ASC (#04-147; Validate for IF)
https://www.citeab.com/antibodies/220072-04-147-anti-asc-antibody-clone-2ei-7
For example, it has been used for IF in PMID: 30054450, dilution 1:100

anti-EEA1 (#3288T; Recommended concentration 1:50 - 1:200, Validate for IF)
https://www.cellsignal.com/products/primary-antibodies/eea1-c45b10-rabbit-mab/3288?srsltid=AfmBOoqa2o8YWxgA4RkZyp62jPb1Yzr4KeJkCJZKtI5Ujvgtla9ArTXf

# Eukaryotic cell lines

Policy information about cell lines and Sex and Gender in Research

| | |
|---|---|
| Cell line source(s) | Jurkat and HK-2 were originally from ATCC.<br>HEK293T and caspase-8-/- HEK293T were from Dr. Hamid Kashkar. |
| Authentication | No authentication. But It has been confirmed in lab by testing phenotypes with positive controls and observing morphologies. |
| Mycoplasma contamination | They were regularly tested in lab and confirmed to be free of contamination. |

| Commonly misidentified lines (See ICLAC register) | *Name any commonly misidentified cell lines used in the study and provide a rationale for their use.* |
|---|---|

## Animals and other research organisms

Policy information about studies involving animals; ARRIVE guidelines recommended for reporting animal research, and Sex and Gender in Research

| | |
|---|---|
| Laboratory animals | C57BL/6J and Nlrp3-/- (C57BL/6J backgound) mice were purchased from Jackson Laboratories (Bar Harbor, ME, USA). Dectin-2-deficient mice (C57BL/6J background) were a kind gift from Yoichiro Iwakura (University of Tokyo, Tokyo, Japan). 6-week-old male and female C57BL/6J mice were purchased for in vivo experiments. All animals were bred in pathogen-free conditions in microisolator cages and were treated according to institutional guidelines following approval by the University of California IACUC. Mice were kept between 22.2°C ± 1.1°C with a humidity of 50-55% and 12-hour lights on/12-hour lights off cycle. CO2 was used to euthanize mice. Bones from Mlkl-/-(C57BL/6 background) and Mlkl-/-Casp8-/- mice (C57BL/6 background) were from Dr. Egil Lien at UMASS Chan Medical School, Wooster, MA, and described previously69. We routinely use male and female mice aged 6-10 weeks for in vitro assays. |
| Wild animals | No wild animals are used in this study |
| Reporting on sex | Both male and female mice were used in the experiments. |
| Field-collected samples | No filed-collected samples are used in this study |
| Ethics oversight | All studies described have been approved by UC Irvine oversight committees, including the use of mice as a source of macrophages and dendritic cells by the UC Irvine IACUC. All systemic infection work was approved by the Institutional Animal Care and Use Committee at the Lundquist Institute for Biomedical Innovation at Harbor-UCLA Medical Center. |

Note that full information on the approval of the study protocol must also be provided in the manuscript.

## Plants

| | |
|---|---|
| Seed stocks | No plants are used in this study. |
| Novel plant genotypes | No plants are used in this study. |
| Authentication | No plants are used in this study. |
